# ConDeTri - A Content Dependent Read Trimmer for Illumina Data

**DOI:** 10.1371/journal.pone.0026314

**Published:** 2011-10-19

**Authors:** Linnéa Smeds, Axel Künstner

**Affiliations:** Department of Evolutionary Biology, Evolutionary Biology Centre, Uppsala University, Uppsala, Sweden; Saint Louis University, United States of America

## Abstract

During the last few years, DNA and RNA sequencing have started to play an increasingly important role in biological and medical applications, especially due to the greater amount of sequencing data yielded from the new sequencing machines and the enormous decrease in sequencing costs. Particularly, Illumina/Solexa sequencing has had an increasing impact on gathering data from model and non-model organisms. However, accurate and easy to use tools for quality filtering have not yet been established. We present ConDeTri, a method for content dependent read trimming for next generation sequencing data using quality scores of each individual base. The main focus of the method is to remove sequencing errors from reads so that sequencing reads can be standardized. Another aspect of the method is to incorporate read trimming in next-generation sequencing data processing and analysis pipelines. It can process single-end and paired-end sequence data of arbitrary length and it is independent from sequencing coverage and user interaction. ConDeTri is able to trim and remove reads with low quality scores to save computational time and memory usage during *de novo* assemblies. Low coverage or large genome sequencing projects will especially gain from trimming reads. The method can easily be incorporated into preprocessing and analysis pipelines for Illumina data.

**Availability and implementation:**

Freely available on the web at http://code.google.com/p/condetri.

## Introduction

Sequencing technologies evolve rapidly. Since Sanger sequencing [1] was introduced, many genomes have been sequenced, including large eukaryotic genomes such as human, mouse and chicken. Recently, several next generation sequencing (NGS) methods have been released and established in biological and medical sciences (see e.g. [2], [3]). However, NGS techniques differ from traditional Sanger sequencing among others with respect to the error probabilities of each read. For Illumina sequencing, the probability of sequencing errors increases exponentially from the 5′ to the 3′ end of a sequencing read [4]. Read accuracy is crucial to consider when using NGS data because it not only affects the assembly and mapping process, but also downstream applications like single nucleotide polymorphism (SNP) discovery and copy number variation (CNV) identification.

Programs that perform *de novo* assemblies of NGS data generally exploit either an overlapping-consensus approach (see for example [5]) or implement a de Bruijn graph [6]. Popular short read *de novo* assemblers like Velvet [7] or SOAPdenovo [8] use the latter approach whereas genome assemblers developed for Sanger or 454 data like Cabog [9] or Newbler [10] use the overlapping-consensus technique. Regardless of the technique used for the assembly, all assembly programs take as input sequenced reads and perform an assembly without a reference genome. Generally, base quality is not used in the contig building of the assembly process. However, programs should use base quality information to correct or to remove erroneous bases from the assembly process in order to reduce the search space. This is especially important for programs based on the de Bruijn graph because it will allow them to save computational resources, thereby reduce assembly time and enabling a more correct assembly.

Correcting for sequencing errors can be done in two different ways. Either bases or reads with low quality are removed completely [11] or erroneous bases are corrected without removing them [12], [13], [14], [15], [16]. The latter approach assumes very high coverage per sequenced base in order to identify erroneous bases, whereas the first approach can be applied to high and low coverage sequence data. Generally, to remove bases (trimming), the quality value for each base is evaluated and bases are removed if they do not exceed a certain quality threshold. This can be done from either end of the read, or along the whole read.

Here, we present our content depend trimming (ConDeTri, available at http://code.google.com/p/condetri) program designed for read trimming of Illumina data. The program removes potential sequencing errors starting from the 3′ read end and also removes reads containing too many low quality bases.

## Results and Discussion

### Test environment

To test the performance of ConDeTri, we used two different *Drosophila melanogaster* data sets obtained by whole genome paired-end sequencing (NCBI SRA:SRR063698 and NCBI SRA:SRR063699) and two different insert sizes (∼170 bp and ∼280 bp) from a *Gallus gallus* resequencing project [17] (NCBI SRA:SRX043655 and NCBI SRA:SRX043656). We used SolexaQA [11] to investigate the quality of the data. Data coming from *D. melanogaster* showed two distinct quality patterns. Generally, data set SRR063698 showed much better and higher Illumina quality scores than the data coming from SRR063699 which makes the two data sets especially useful to test the influence of trimming on ‘good’ and ‘bad’ sequencing data. For both sets we also prepared a reduced version, where we selected 25% of the paired-end reads randomly (below referred to as the reduced data or reduced set). Overall, the *G. gallus* data showed high Illumina quality scores. We did not create a reduced set from this data. Additionally, we used data from the collared flycatcher (*Ficedula albicollis*) genome-sequencing project to test the performance of ConDeTri on a non-model species where no genome sequence is available.

Sequencing reads can be duplicated due to biased PCR replication, sequencing artifacts, and genomic DNA shearing at the same location in different DNA-molecules [18], [19], [20], [21]. Therefore, we scanned each data set for duplicated paired-end reads using the first 50 nucleotides in each read and kept only one unique read-pair. Additional read-pairs were removed. We removed around 3% of all read-pairs in the SRR063698 data. Less than 1% of all read-pairs were removed in the SRR063699, SRX043655, and SRX043656 data. Raw data and filtered data are summarized in Table 1.

**Table 1:** Data.

Data	Reads	
	unfiltered	filtered
SRR063698	55,932,362	54,256,212
SRR063698 reduced	13,983,090	13,705,644
SRR063699	27,021,832	26,788,696
SRR063699 reduced	6,755,458	6,721,328
SRX043655	224,522,574	223,836,804
SRX043656	250,789,142	248,749,514

Amount of raw reads (unfiltered) and reads after filtering for PCR duplicates.

We tested ConDeTri against untrimmed data, and against three recently published methods, SolexaQA version 1.7 [11], the Bwa quality trimming algorithm [22] as implemented in SolexaQA version 1.7 and Quake version 0.2 [13] using the *D. melanogaster* and *G. gallus* data. SolexaQA and Bwa quality trimming use a quality based read trimming approach whereas Quake performs quality detection and correction of potential sequencing errors. For the three trimming programs we used same parameters for quality cutoff and minimum read length to be able to make a fair comparison between the programs. Quality cutoff was set to 25 and minimum read length to 50. The values were chosen after inspecting several data sets (see Method section). SolexaQA and Bwa have no other relevant parameters that can be adjusted to improve filtering quality. For each data set, the optimal *k* parameter according to the genome size was calculated to be able to run count-qmers from Quake. Quake could not be run without user interaction because the data does not provide enough sequencing depth to estimate the coverage cutoff parameter. Therefore, we investigated the coverage histograms for each data set manually and chose the best cutoff value according to the Quake online manual.

Data that was trimmed based on quality and untrimmed was *de novo* assembled using SOAPdenovo version 1.04 [8] with k-mer sizes ranging from 19 to 31. For each method and data set, the best assembly was chosen according to the N50 size of the assembly and then aligned to the *D. melanogaster* reference genome (Release 5) or *G. gallus* reference genome (Release WUGSC 2.1/galGal3) using NUCmer version 3.07 (64-bit compiled version) from the MUMmer package [23]. To infer the alignment quality, we used show-tiling from the MUMmer package with a minimum percent identity of 95% to construct a tiling path out of the query scaffolds as mapped to the reference sequences (recall rate) and we estimated the proportion of the assembly that could be aligned onto the reference genome (accuracy rate). Single nucleotide polymorphism (SNP) frequency per base was estimated as follows. First, reads were mapped onto the *G. gallus* reference genome using Bwa version 0.5.9 [24]. Second, SNP calling was done applying the pileup command as implemented in SAMtools version 0.1.16 [25]. The coverage cutoff was set to 60 after inspecting the coverage across the genome to avoid false positive SNP calls due to unresolved repeats. Note, for estimating SNP frequencies we took only the covered genome into account and disregarded the proportion that was not covered by reads.

### Trimming effect

Theoretically, trimming should reduce the problem complexity of *de novo* assemblies since it shrinks the size of the de Bruijn graph. This should lead to more accurate *de novo* assemblies. Still, there are no measures available to quantify the quality of a *de novo* assembly. Therefore, we estimated the proportion of the assembly that could be mapped to the reference genome (accuracy) and the proportion of the genome that was covered with the assembly (recall). We think that accuracy is more important than recall because it is gives the amount of the assembly that is correctly assembled.

**Figure 1:**
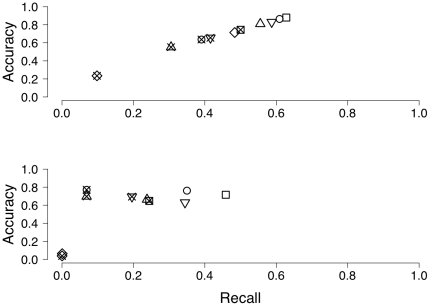
Recall and accuracy for different data sets. The proportion of the genome covered (recall) and the proportion of the assembly mapped onto the genome (accuracy) for untrimmed reads (squares), ConDeTri (circles), Bwa (triangle point down), SolexaQA (triangle point up) and Quake (diamonds). Open symbols denote the full data set, and crossed symbols reduced data. The upper panel shows results for SRR0063698, the lower panel for SRR0063699.

For the ‘higher quality’ *D. melanogaster* data set (NCBI SRA:SRR063698) the assembly using no trimming yielded the best accuracy (88%) and the best recall (63%, 106.0 MB of the genome covered). ConDeTri gave quite similar results, with an accuracy of 86% and recall of 61% (102.8 MB of the genome covered). Data filtered using Bwa, SolexaQA or Quake gave slightly less accurate assemblies (83%, 81% and 71%) and also smaller proportions of the genome assembled (recall 59%, 56% and 48%, respectively). Interestingly, the assembly with the longest N50 sizes (Quake) gave the smallest genome assembly. Potentially, mis-assemblies are more common in assemblies with longer N50 sizes. Small assembly mistakes tend to produce continuous sequences from contigs that are not actually located close to each other and can thereby greatly reduce the ability of programs to create longer scaffolds. Using the reduced data showed even more pronounced results in favor of the untrimmed assembly (see Fig. 1 and Table 2).

**Table 2:** Comparison of different filtering strategies for *D. melanogaster*.

Method	Data		Assembly size (Mb)	Assembled genome (Mb)	Accuracy	Recall	N50	Reads (million)
Untrimmed	SRR063698	full	120.5	106.0	0.88	0.63	6,939	54.3
		reduced	113.4	84.4	0.74	0.50	1,717	13.7
	SRR063699	full	108.1	77.4	0.72	0.45	1,278	26.8
		reduced	63.6	41.2	0.65	0.24	411	6.7
ConDeTri	SRR063698	full	118.9	102,8	0.86	0.61	8,222	43.4
		reduced	103.6	65.9	0.64	0.39	1,007	11.0
	SRR063699	full	77.5	59.0	0.76	0.35	508	10.0
		reduced	23.4	18.0	0.77	0.11	234	2.5
SolexaQA	SRR063698	full	115.7	93.7	0.81	0.56	5,691	37.8
		reduced	93.4	51.5	0.55	0.31	677	9.6
	SRR063699	full	60.5	40.2	0.66	0.24	408	8.7
		reduced	16.7	11.6	0.70	0.07	203	2.2
Quake	SRR063698	full	114.3	81.6	0.71	0.48	21,615	49.6
		reduced	70.6	16.5	0.23	0.10	683	10.4
	SRR063699	full	1.9	0.1	0.06	0.00	6,678	5.9
		reduced	1.4	0.1	0.04	0.00	3,808	1.3
Bwa	SRR063698	full	119.4	99.1	0.83	0.59	13,953	46.2
		reduced	107.0	70.1	0.66	0.42	1,264	11.7
	SRR063699	full	92.2	58.1	0.63	0.34	732	17.4
		reduced	47.5	33.0	0.70	0.20	294	4.4

Assembly size, size of assembled *D. melanogaster* reference genome, recall, accuracy, N50 size and number of reads using the different data sets.

The data from the ‘lower quality’ *D. melanogaster* sample (NCBI SRA:SRR063699) gave slightly different results. As the overall quality of the sample was not very good, a smaller proportion of the genome could be assembled. The proportion of the assembly that could accurately mapped onto the reference genome (accuracy) varies between 76% using ConDeTri for trimming and 6% using Quake. Accuracy for the untrimmed data was 72%, 66% for SolexaQA, and 63% for Bwa trimmed data, respectively. Recall was highest in the untrimmed data (45%), followed by ConDeTri (35%), Bwa (34%) and SolexaQA (24%). Quake had the lowest recall (<1%). Interestingly, using only 25% of the data gave a slightly larger proportion of the assembly that was accurate for ConDeTri (77%), Bwa (70%) and SolexaQA (70%) but the overall assembled size of the genome drops down. Untrimmed data gave an accuracy of 65%. Recall was highest using the untrimmed data (24%) reflecting that this data set contains most of the data. Bwa, ConDeTri and SolexaQA have a recall of 20%, 11% and 7% respectively. Again, the assembled data from Quake covered less than 1% of the reference genome. Quake was outperformed using the ‘lower quality’ sample by the read trimming method because the coverage in this sample is not high enough to perform a read correction. According to the authors, coverage should be at least 15× [13]. All results are summarized in Figure 1 and Table 2.

Assembly results for the *G. gallus* data were quite similar between the different trimming methods and the untrimmed data. Accuracy for the read trimming methods ranged from 78% using Bwa to 82% using ConDeTri whereas the untrimmed data gave the most accurate result with 85%. Recall was highest for untrimmed data (82%), followed by ConDeTri (78%), SolexaQA (77%) and Bwa (75%). Interestingly, ConDeTri was the method that removes the largest amount of reads but it gives the best result among the trimming methods. We think untrimmed data gave the best result in this comparison because the assembly has the lowest N50, which potentially reduces the probability of wrongly assembled regions. Additionally, untrimmed data needed more time and memory for the assembly. Especially the latter point can be crucial for sequencing projects. We were not able to retrieve results using Quake on this data set, as it was not able to correct the data from SRX043656. We managed to correct data from SRX043655 but after read correction less than 5% of the original reads were included in the corrected Fastq files. Results for the *G. gallus* data are summarized in Table 3.

**Table 3:** Comparison of different filtering strategies for *G. gallus*.

Method	Assembly size (Mb)	Assembled genome (Mb)	Accuracy	Recall	N50	Reads (million)
Untrimmed	995.2	844.70	0.85	0.82	12,773	472.6
ConDeTri	990.4	808.10	0.82	0.78	26,964	410.9
SolexaQA	988.9	791.40	0.80	0.77	28,907	418.1
Bwa	997.2	778.78	0.78	0.75	32,973	449.6

Assembly size, size of assembled *G. gallus* reference genome, recall, accuracy, N50 size and number of reads using the different data sets.

We also applied a SNP calling method on the *G. gallus* data set to be able to compare the performance of read trimming versus untrimmed reads. As the individual used for sequencing and re-sequencing the *G. gallus* genome is highly inbred [17], [26] we expect a much lower heterozygosity rate than in natural populations and that the per base SNP frequency is lower for trimmed data than for untrimmed data because the untrimmed data contains more sequencing errors. We found one SNP every 1,299 bp in the untrimmed data and one every ∼1,450 in the trimmed data sets, regardless which trimming method was applied, which was consistent with our predictions. Note that the SNP frequency per base is much lower than the estimated frequency of one SNP in 374 bp [26] in *G. gallus*. We think that the difference between trimmed and untrimmed data is mainly based on sequencing errors. Per base coverage in the data ranged from 39.5× in the untrimmed data to 33.2× in the data using SolexaQA for trimming. Data trimmed using Bwa had almost the same coverage per base as the untrimmed data (37.3×) whereas ConDeTri data has coverage of 35.9×. The differences in per base coverage between the different trimmed and the untrimmed data corresponds 94%, 91% and 84% for Bwa, ConDeTri and SolexaQA, respectively. As shown before, SNP frequency was almost identical in the trimmed data sets. We conclude that coverage has not a strong impact on SNP calling in our data. Furthermore, we think the difference in SNP frequency between trimmed and untrimmed data, is mainly due to the presence of more sequencing errors in the untrimmed data but more sophisticated tests are needed to verify this findings, which is outside the scope of this study.

To test the effect of filtering on a non-model organism where no reference genome is available, we made use of data from an attempt towards genome sequencing in the collared flycatcher (H. Ellegren et al. unpublished), a small songbird. Although the genome size of this species has not been determined, there is a high degree of genome size conservation among birds with most song birds having an estimated haploid DNA content of 1.1–1.3 Gb [27]. We cannot calculate recall and accuracy for this data set because there is no reference genome available for the collared flycatcher. Therefore, we concentrated on assembly time and memory usage because this should be related to the complexity of the de Bruijn graph. We selected 4 lanes of Illumina Genome Analyzer II data (insert size ∼200 bp), which gave a total of 19.9 Gb of untrimmed sequence data. After trimming using ConDeTri, 15.6 Gb sequence data remained for the assembly. We tested only untrimmed data and ConDeTri on this data, as they were the best performers on the *D. melanogaster* and *G. gallus* data. Assembly size and N50 size was quite similar between trimmed and untrimmed data (not shown) but running time for the trimmed data set was 152 minutes with a peak memory usage of 39 GB whereas the untrimmed finished within 388 min and a peak memory usage of 76 GB of RAM showing the big impact of sequencing errors on memory usage and running time. Given that the untrimmed data contains around one third more data, the running time is more than twice as long and the memory usage almost doubles in comparison to the trimmed data.

Trimming may not have a big impact on *de novo* assemblies for smaller genomes, because there is a sufficient amount of per base coverage. Also, the de Bruijn graph is still quite small for genomes about the size of the *D. melanogaster* data sets that we used so that there is little benefit of trimming reads on the assembly. However, we have shown that trimming has an effect on the assembly process of larger genomes with a more complex genome structure (higher repeat content, and higher proportion of non-coding sequences). Using untrimmed data for the assembly of e.g. mammalian or avian genomes, where genomes sizes exceed 1 Gb, complicates the de Bruijn graph. Sequencing errors introduce k-mers in the graph that do not occur frequently, which increases the number of nodes and edges and can make the graph unwieldy even for powerful computers. One approach to avoid this is to correct for erroneous k-mers, as programs like Quake does. However, this is only possible if a sufficient base coverage is reached to be able to correct for sequencing errors. We have shown that the data we have used does not provide enough coverage to be able to correct sequencing errors using a k-mer based approach. Instead it is better to remove bases or reads that do not fulfill a certain quality criteria.

## Methods

### Implementation

ConDeTri is implemented in Perl (required version 5.8.9 or above), is platform independent, has no additional hardware or library requirements, and is distributed under Artistic License/GPL. It is designed to run single-threaded on desktop computers or on cluster machines. In default mode, it can be run by giving only one Fastq file for single-end sequencing or two Fastq files for paired-end sequencing. More advanced options allow the user to control such things as the quality values used for trimming, trimming size, the fraction of a read containing high quality bases, and the quality format used (either Illumina/Solexa Fastq format or Sanger Fastq format is chosen by different offset scores).

Our trimming approach does not correct the actual quality scores called by the Illumina pipeline. Instead, it removes bases with quality values lower than a threshold from the 3′-end of a read and checks the remaining read for internal low quality bases.

ConDeTri applies two filtering steps on each read. First, each read is trimmed, one base at the time, in an iterative process. Starting from the 3′-end of the read, bases are removed if the corresponding quality score is lower than a threshold *Q_H_*. When reaching a base with a quality score higher than *Q_H_*, the base is kept temporarily while following bases are evaluated. After parsing a certain number of consecutive high quality bases, *n_H_*, the trimming is terminated. However, even bases with low quality scores below *Q_H_*, recorded before *n_H_* is reached, are saved temporarily. Up to *n_L_* consecutive low quality bases are accepted when they are surrounded by high quality bases. If *n_L_* is overrun, all temporarily saved bases are removed, and the process starts over again. The trimming continues until either *n_H_* consecutive high quality bases are found, or the read is trimmed down to length *L*.

For a trimmed read to be approved, it must contain more than a certain fraction *f* of bases with a quality score higher than *Q_H_*, and no bases with a quality score less than a lower bound threshold *Q_L_*. If a base has a quality score lower than *Q_L_* the read is removed. When all reads have been trimmed, each read or each read pair is examined. If a single read passes the quality check, it is stored in a new Fastq file. For paired end reads, pairs where both the reads fulfill the quality demands are saved in new paired Fastq files. If a pair contains only one read passing the quality requirements, the high quality read is saved in an extra Fastq. These reads can be used as single end reads. Besides Fastq files, ConDeTri reports the number of scanned and removed reads and the number of reads that are present as paired-end and as single-end reads. Figure S1 summarizes the algorithm in a flowchart and Figure S2 gives two examples.

Per default, the high quality score (*Q_H_*) is set to 25, which is similar to a sequencing error probability of 0.0032. This value was chosen after inspecting quality score distributions from several data sets with different insert sizes from the collared flycatcher genome-sequencing project, as a level where the number of bases kept are of highest possible quality without having a considerable loss of reads. For the sets inspected, changing the quality threshold to 30 resulted in a loss of the majority of reads during filtering. On the other hand, lowering it to 20 did not increase the number of reads kept significantly, but the per base error probability of those reads will be up to three times higher (∼0.01). However, the default value is by no means universal, and the threshold should be set according to the data. The low quality score (*Q_L_*) is set to 10, which equals a probability of a sequencing error of 0.0909, the fraction *f* of bases with a quality score higher than *Q_H_* is set to 80% and *L*, the minimum number of bases after trimming, is set to 50, to prevent saving reads that are too short for *de novo* assembly. The parameters *n_H_* and *n_L_* are set to 5 and 1, respectively. This means that for each low quality-base there must be at least five high quality bases, which is more than the *Q_H_ value of* 80%. The connection between these numbers must be considered when tweaking the parameters – keeping *n_H_* and *n_L_* as 5 and 1 but increasing the *Q_H_ to 95*% results in removing a large proportion of reads in the second step. However, all these settings can be changed as desired. Quality score distribution along reads and read length distribution after trimming for the libraries used for choosing the default values are shown in Figures S3, S4, S5, S6, S7, S8, S9, S10, S11, S12, S13, S14, S15, S16, S17 and Table S1. ConDeTri can read all three different Fastq quality score standards: Illumina and Solexa (early Illumina) quality scores with an offset of 64 and Sanger standard with an offset of 33.

### Conclusion

The main focus of our quality filtering approach was to provide an accurate, standardized and easy to use method for trimming Illumina sequencing data. In comparison to other programs, data filtered with ConDeTri gave better results with respect to the size of the assembled data and also the accuracy of the *de novo* assembly. In comparison to untrimmed data, less memory and time is needed for *de novo* assemblies. This is crucial for larger eukaryotic genomes, because affordable computational resources are still a limiting factor in performing assemblies of larger genomes using several insert sizes for paired-end sequencing. Using quality-filtered data reduces the de Bruijn graph in the assembly process and should improve downstream analyses of NGS data (e.g. SNP calling).

## Supporting Information

Figure S1 **Flowchart ConDeTri.** Flowchart for the ConDeTri algorithm for read trimming.
(PDF)

Figure S2 **Examples of read trimming.** Two examples of read trimming using ConDeTri.
(PDF)

Figure S3 **Figures S3, S4, S5, S6, S7, S8, S9 – Quality plots for forward reads.** Examples of quality plots for the forward read in paired-end Illumina sequencing from the collared flycatcher genome-sequencing project. Four libraries of different insert sizes were run in several lanes each, distributed over five flowcells (flowcell 1–3 was run on a GAII, flowcell 4–5 on a HiSeq2000), only a subset of the plots is shown here. The solid red line in bold shows quality score 25, the default settings for *Q_H_*. The thinner solid line shows the default minimum quality *Q_L_* = 10, and the blue vertical dashed line shows the default minimum allowed read length 50 bp. The two dashed red lines shows quality scores 30 and 20 respectively. The corresponding backward reads are shown in Figure S10, S11, S12, S13, S14, S15, S16.
(PNG)

Figure S4 (PNG)

Figure S5 (PNG)

Figure S6 (PNG)

Figure S7 (PNG)

Figure S8 (PNG)

Figure S9 (PNG)

Figure S10 **Figures S10, S11, S12, S13, S14, S15, S16– Quality plots for backward reads.** The backward reads corresponding to Figure S3, S4, S5, S6, S7, S8, S9. For the first flow cell, only 65 bp were sequenced for the backward reads due to technical problems.
(PNG)

Figure S11 (PNG)

Figure S12 (PNG)

Figure S13 (PNG)

Figure S14 (PNG)

Figure S15 (PNG)

Figure S16 (PNG)

Figure S17 **Example length distribution after trimming.** Example of read length distribution for the filtered data set corresponding to Figure S3. A majority of the reads are kept at full length. The wave-like pattern in cycles of 5 bp comes from that *n_H_* is set to 5.
(PNG)

Table S1 **Data before and after filtering.** The number of reads before and after filtering for the data used for estimating ConDeTri default parameters.
(PDF)

## References

[1] Sanger F, Nicklen S, Coulson AR (1977). DNA sequencing with chain-terminating inhibitors.. Proc Natl Acad Sci USA.

[2] Mardis ER (2008). The impact of next-generation sequencing technology on genetics.. Trends in Genetics.

[3] Metzker ML (2010). Sequencing technologies - the next generation.. Nat Rev Genet.

[4] Dohm JC, Lottaz C, Borodina T, Himmelbauer H (2008). Substantial biases in ultra-short read data sets from high-throughput DNA sequencing.. Nucleic Acids Research.

[5] Scheibye-Alsing K, Hoffmann S, Frankel A, Jensen P, Stadler PF (2009). Sequence assembly.. Computational Biology and Chemistry.

[6] Pevzner P, Tang H, Waterman M (2001). An Eulerian path approach to DNA fragment assembly.. PNAS.

[7] Zerbino D, Birney E (2008). Velvet: Algorithms for De Novo Short Read Assembly Using De Bruijn Graphs.. Genome Research.

[8] Li R, Zhu H, Ruan J, Qian W, Fang X (2010). De novo assembly of human genomes with massively parallel short read sequencing.. Genome Research.

[9] Miller JR, Delcher AL, Koren S, Venter E, Walenz BP (2008). Aggressive assembly of pyrosequencing reads with mates.. Bioinformatics.

[10] Margulies M, Egholm M, Altman WE, Attiya S, Bader JS (2005). Genome sequencing in microfabricated high-density picolitre reactors.. Nature.

[11] Cox MP, Peterson DA, Biggs PJ (2010). SolexaQA: At-a-glance quality assessment of Illumina second-generation sequencing data.. BMC Bioinformatics.

[12] Dohm JC, Lottaz C, Borodina T, Himmelbauer H (2007). SHARCGS, a fast and highly accurate short-read assembly algorithm for de novo genomic sequencing.. Genome Research.

[13] Kelley DR, Schatz MC, Salzberg SL (2010). Quake: quality-aware detection and correction of sequencing errors.. Genome Biology.

[14] Salmela L (2010). Correction of sequencing errors in a mixed set of reads.. Bioinformatics.

[15] Schröder J, Schröder H, Puglisi SJ, Sinha R, Schmidt B (2009). SHREC: a short-read error correction method.. Bioinformatics.

[16] Ilie L, Fazayeli F, Ilie S (2011). HiTEC: accurate error correction in high-throughput sequencing data.. Bioinformatics.

[17] Ye L, Hillier LW, Minx P, Thane N, Locke D (2011). A vertebrate case study of the quality of assemblies derived from next-generation sequences.. Genome Biology.

[18] Kozarewa I, Ning Z, Quail MA, Sanders MJ, Berriman M (2009). Amplification-free Illumina sequencing-library preparation facilitates improved mapping and assembly of (G+C)-biased genomes.. Nature Methods.

[19] Li R, Fan W, Tian G, Zhu H, He L (2010). The sequence and de novo assembly of the giant panda genome.. Nature.

[20] Miller J, Koren S, Sutton G (2010). Assembly algorithms for next-generation sequencing data.. Genomics.

[21] Ratan A, Zhang Y, Hayes VM, Schuster SC, Miller W (2010). Calling SNPs without a reference sequence.. BMC Bioinformatics.

[22] Bwa website.. http://bio-bwa.sourceforge.net/.

[23] Kurtz S, Phillippy A, Delcher A, Smoot M, Shumway M (2004). Versatile and open software for comparing large genomes.. Genome Biology.

[24] Li H, Durbin R (2009). Fast and accurate short read alignment with Burrows-Wheeler transform.. Bioinformatics (Oxford, England).

[25] Li H, Handsaker B, Wysoker A, Fennell T, Ruan J (2009). The Sequence Alignment/Map format and SAMtools.. Bioinformatics (Oxford, England).

[26] ICGC (2004). A genetic variation map for chicken with 2.8 million single-nucleotide polymorphisms.. Nature.

[27] Gregory TR (2005). Synergy between sequence and size in Large-scale genomics.. Nat Rev Genet.

