# Supplementary figures and images for: ConDeTri - A Content Dependent Read Trimmer for Illumina Data
Source: PLoS One. 2011 Oct 19;6(10):e26314. doi: 10.1371/journal.pone.0026314 (PMC3198461; doi:10.1371/journal.pone.0026314)

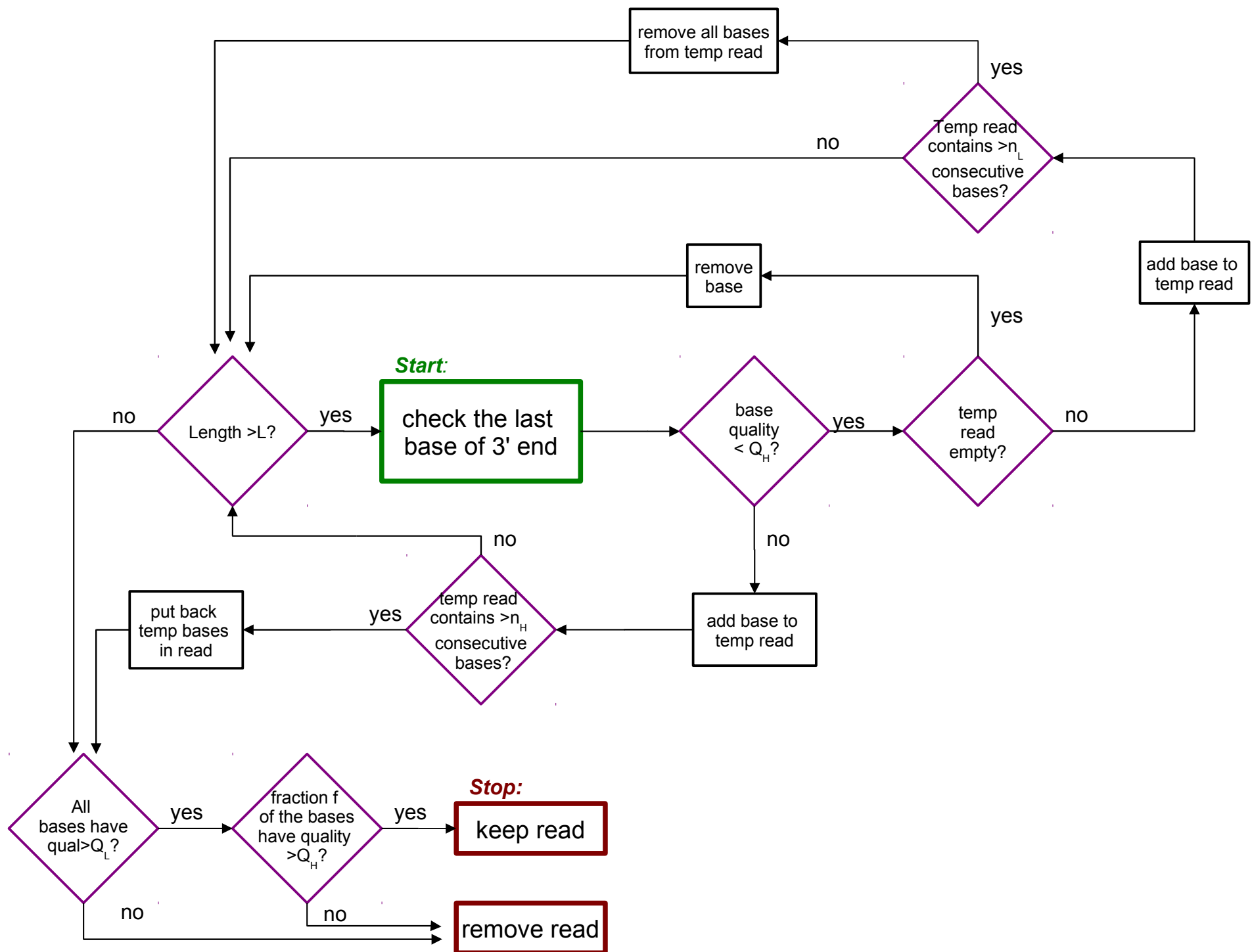

Supplement: Figure S1 — Flowchart ConDeTri. Flowchart for the ConDeTri algorithm for read trimming. (PDF) [file pone.0026314.s001.pdf]

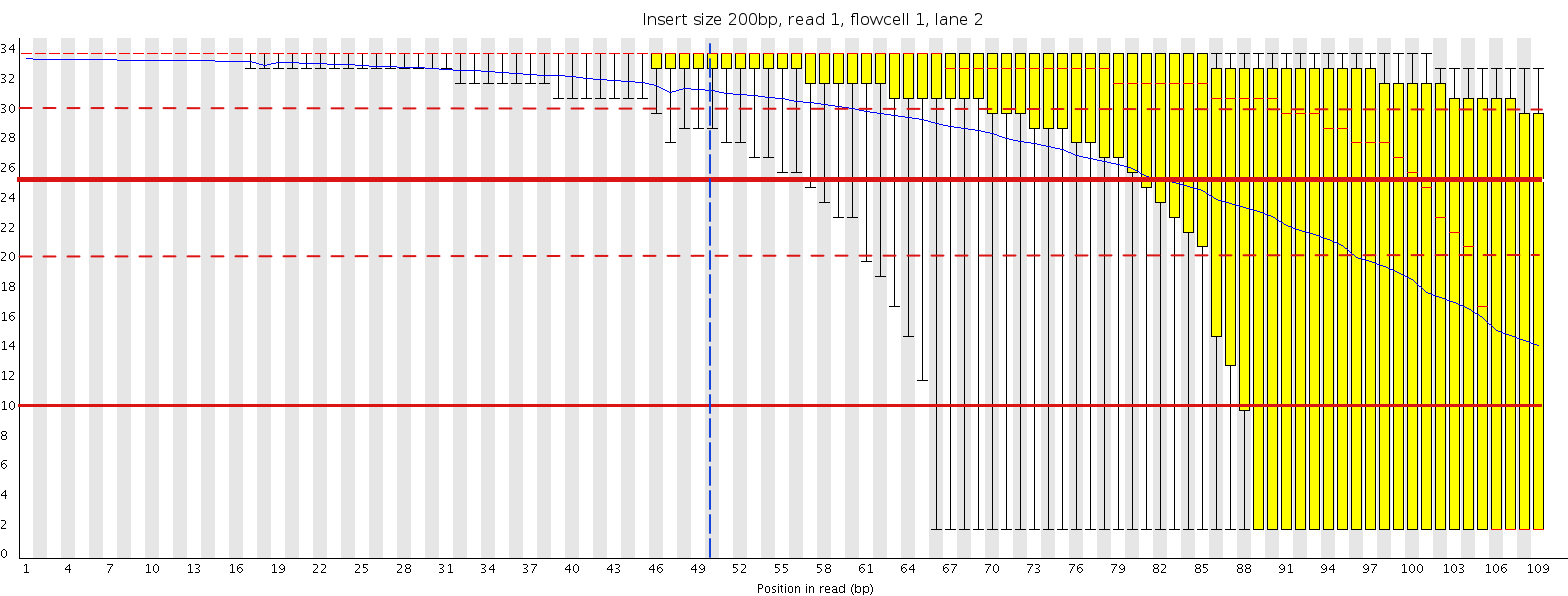

Supplement: Figure S3 — Figures S3, S4, S5, S6, S7, S8, S9 – Quality plots for forward reads. Examples of quality plots for the forward read in paired-end Illumina sequencing from the collared flycatcher genome-sequencing project. Four libraries of different insert sizes were run in several lanes each, distributed over five flowcells (flowcell 1–3 was run on a GAII, flowcell 4–5 on a HiSeq2000), only a subset of the plots is shown here. The solid red line in bold shows quality score 25, the default settings for QH. The thinner solid line shows the default minimum quality QL = 10, and the blue vertical dashed line shows the default minimum allowed read length 50 bp. The two dashed red lines shows quality scores 30 and 20 respectively. The corresponding backward reads are shown in Figure S10, S11, S12, S13, S14, S15, S16. (PNG) [file pone.0026314.s003.png]

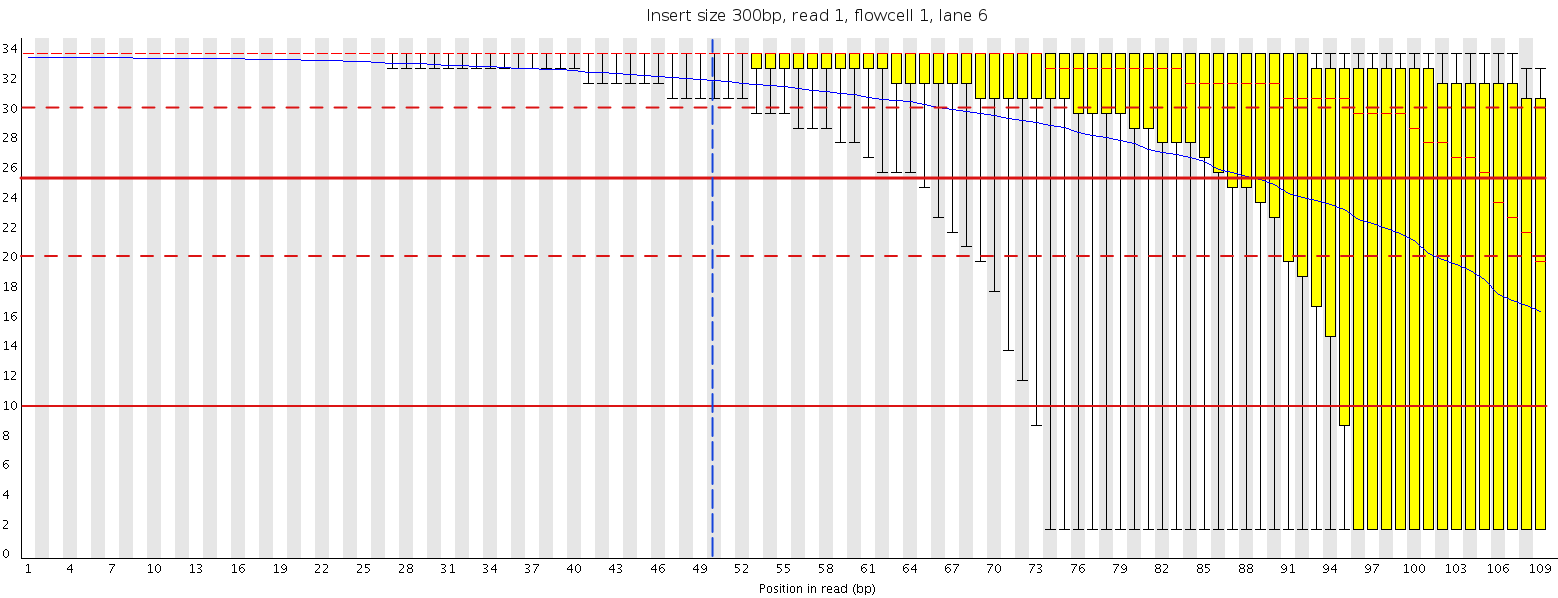

Supplement: Figure S4 — (PNG) [file pone.0026314.s004.png]

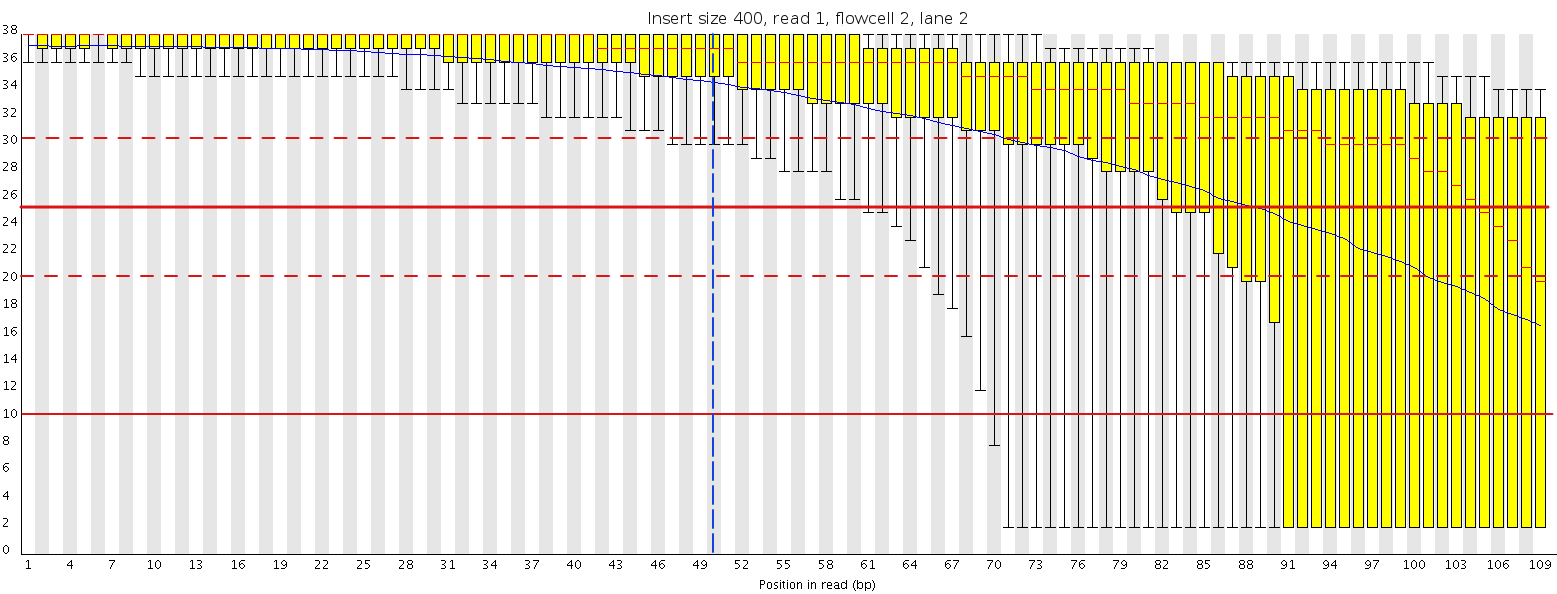

Supplement: Figure S5 — (PNG) [file pone.0026314.s005.png]

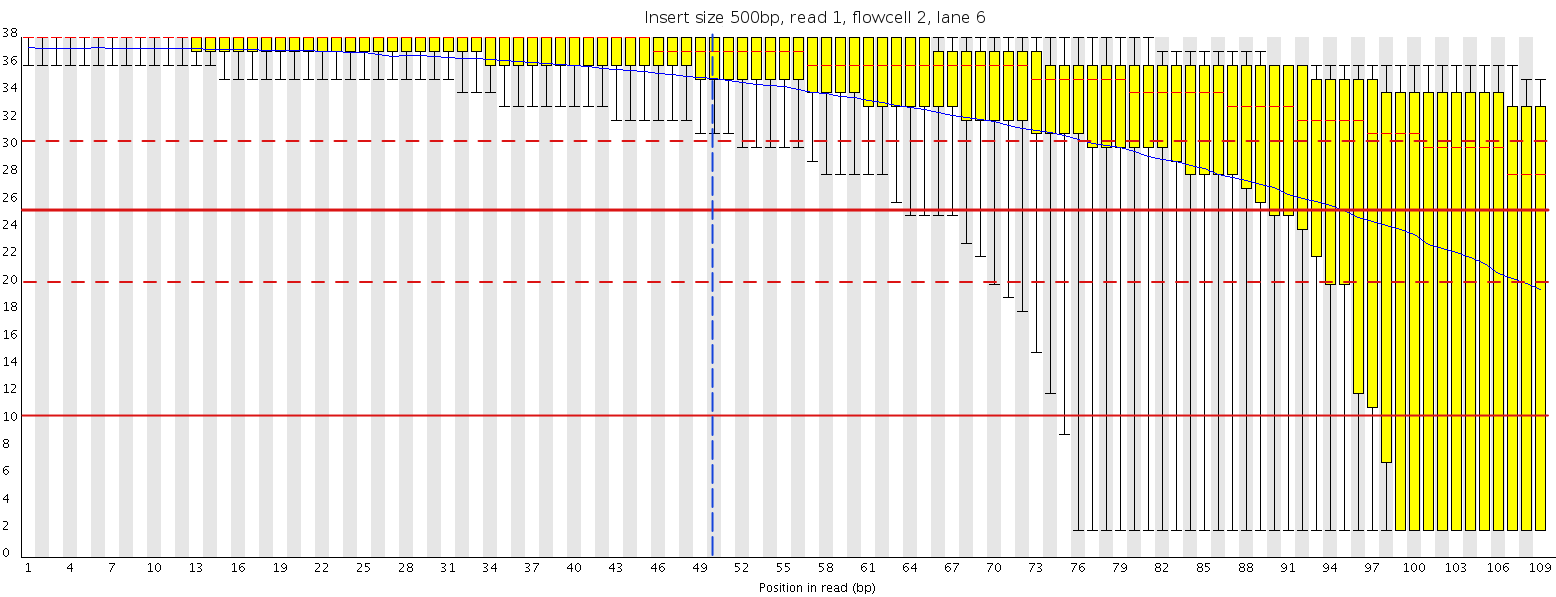

Supplement: Figure S6 — (PNG) [file pone.0026314.s006.png]

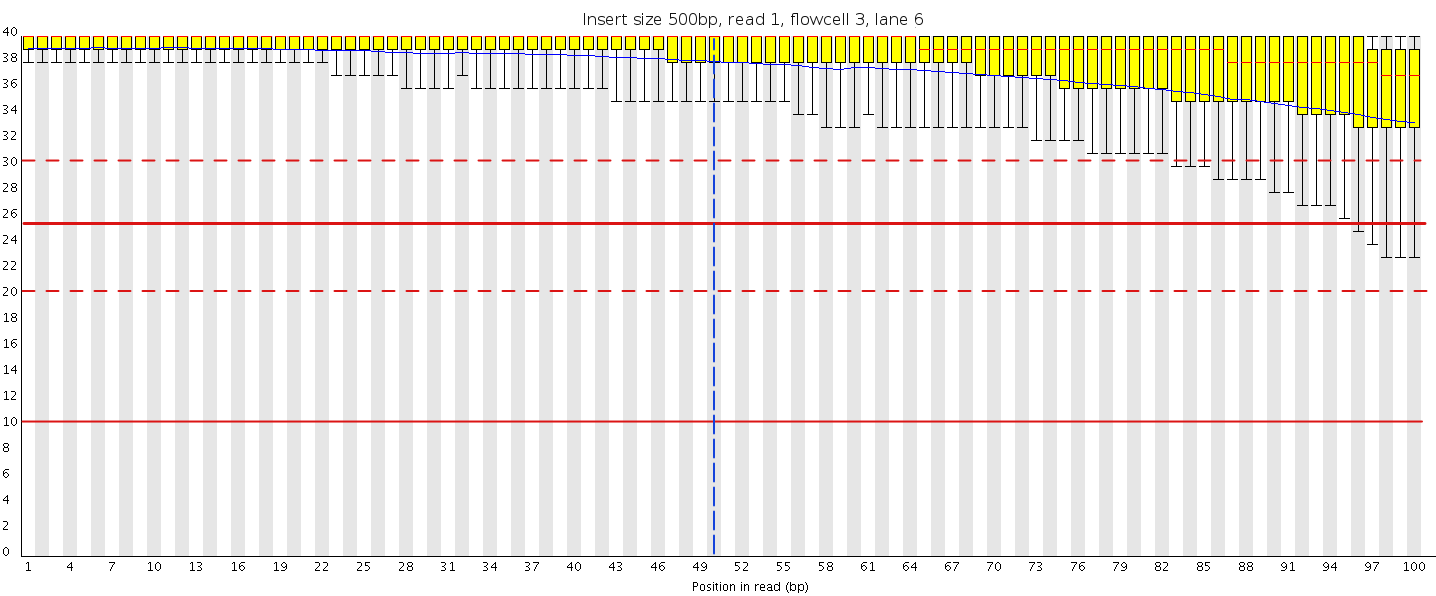

Supplement: Figure S7 — (PNG) [file pone.0026314.s007.png]

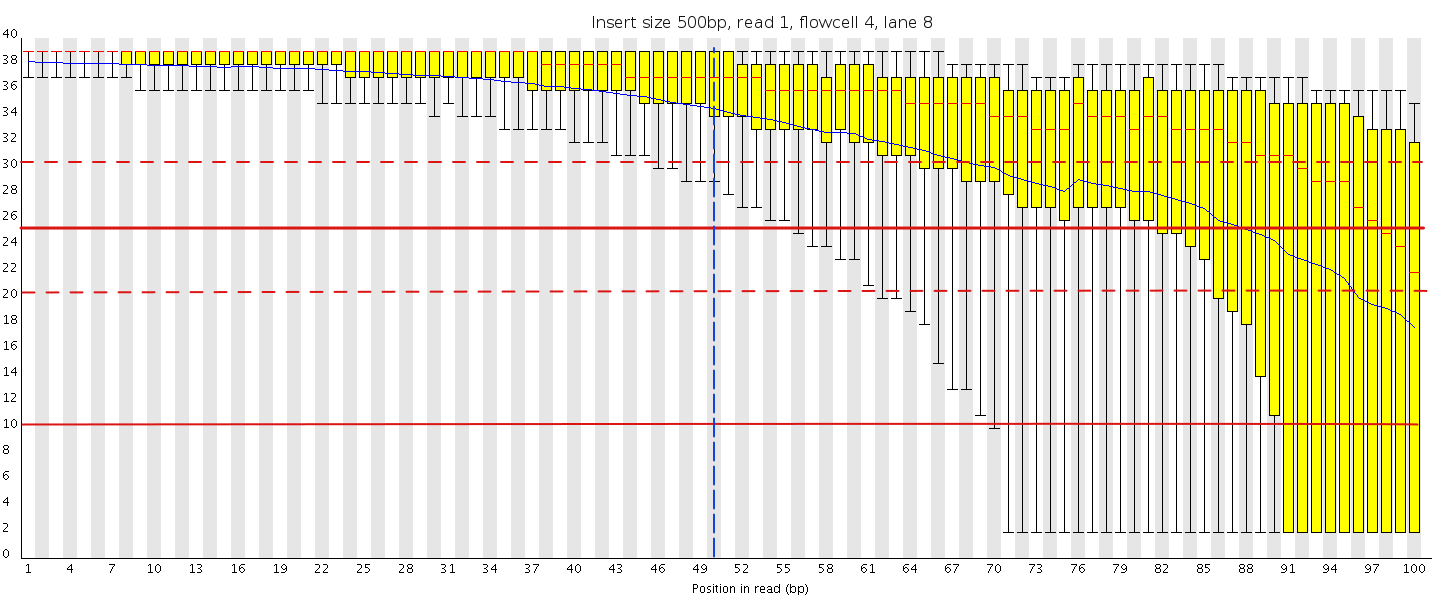

Supplement: Figure S8 — (PNG) [file pone.0026314.s008.png]

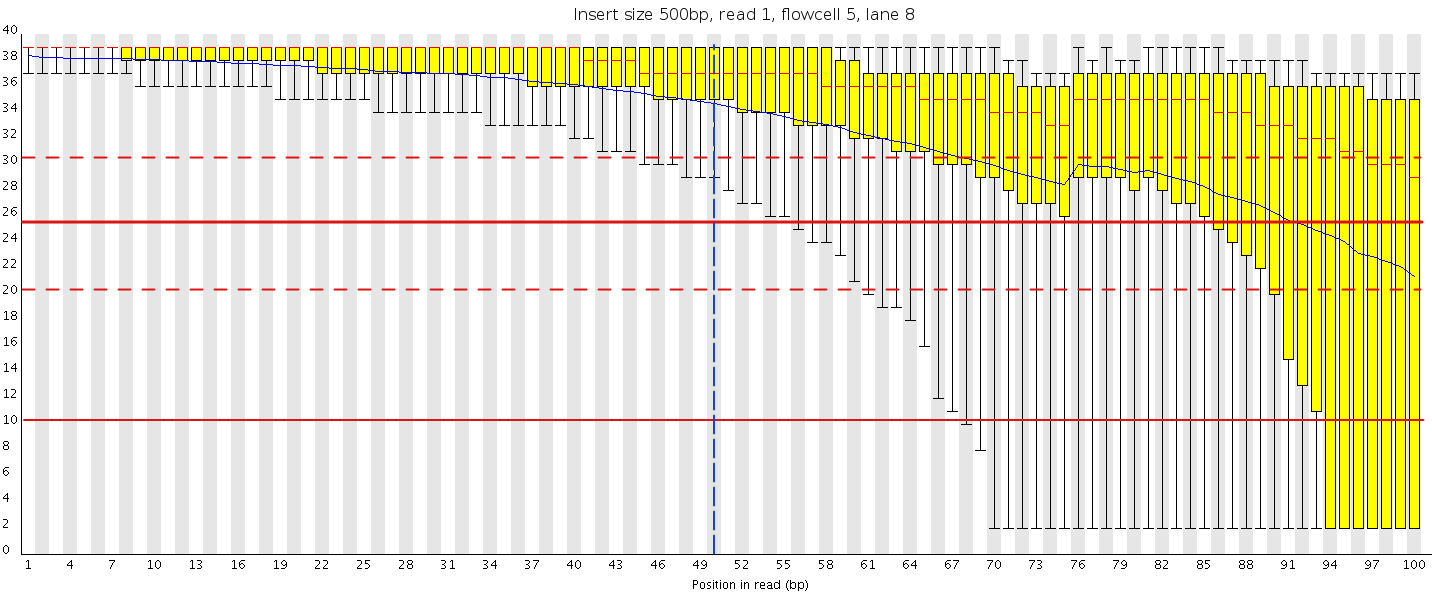

Supplement: Figure S9 — (PNG) [file pone.0026314.s009.png]

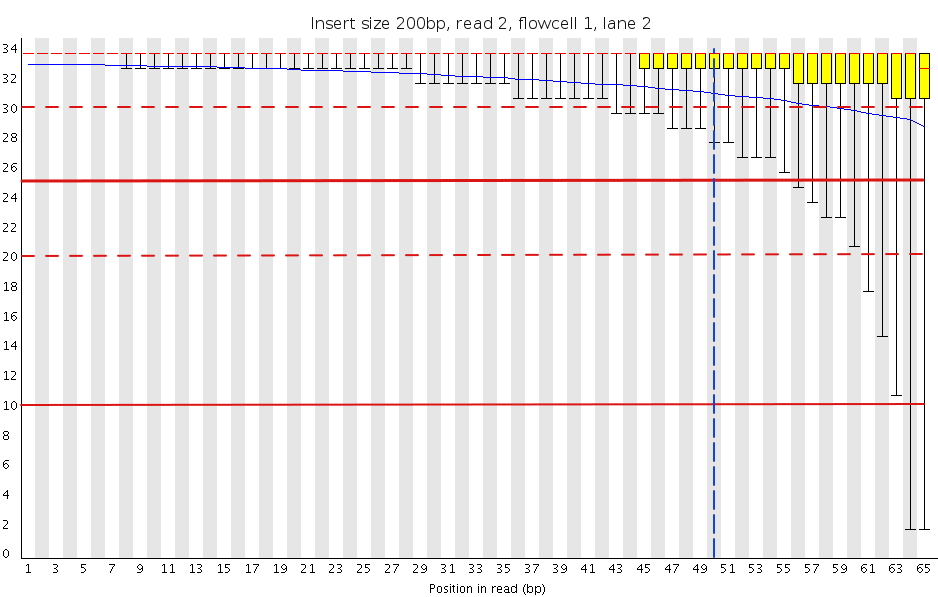

Supplement: Figure S10 — Figures S10, S11, S12, S13, S14, S15, S16– Quality plots for backward reads. The backward reads corresponding to Figure S3, S4, S5, S6, S7, S8, S9. For the first flow cell, only 65 bp were sequenced for the backward reads due to technical problems. (PNG) [file pone.0026314.s010.png]

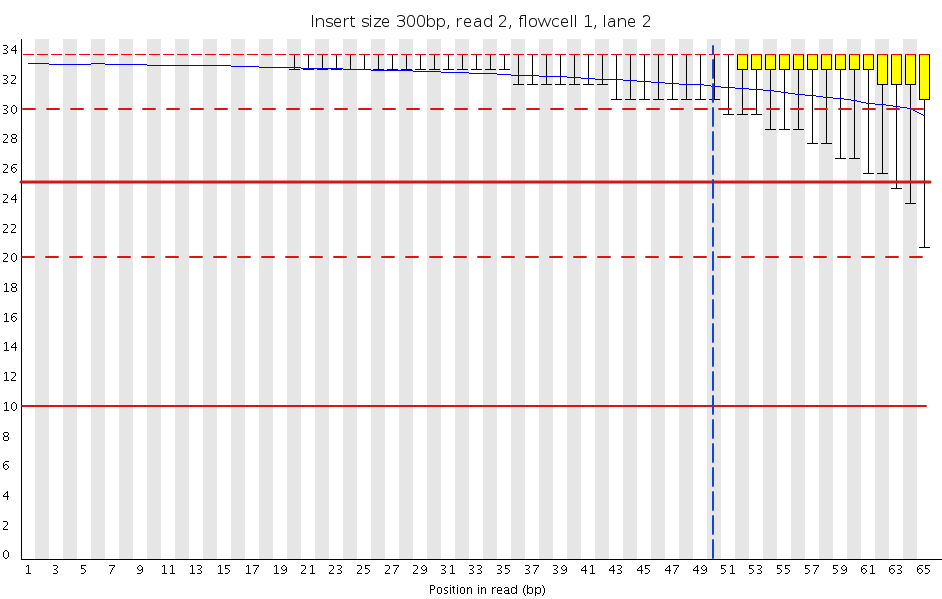

Supplement: Figure S11 — (PNG) [file pone.0026314.s011.png]

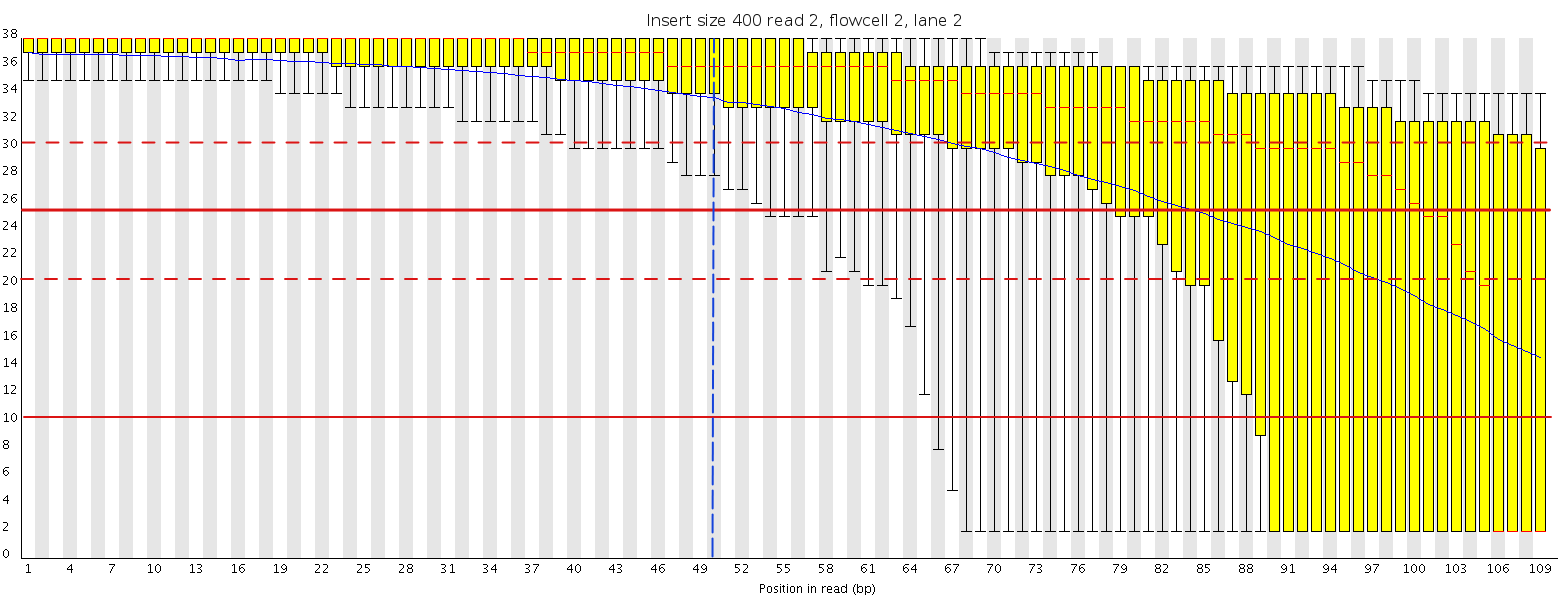

Supplement: Figure S12 — (PNG) [file pone.0026314.s012.png]

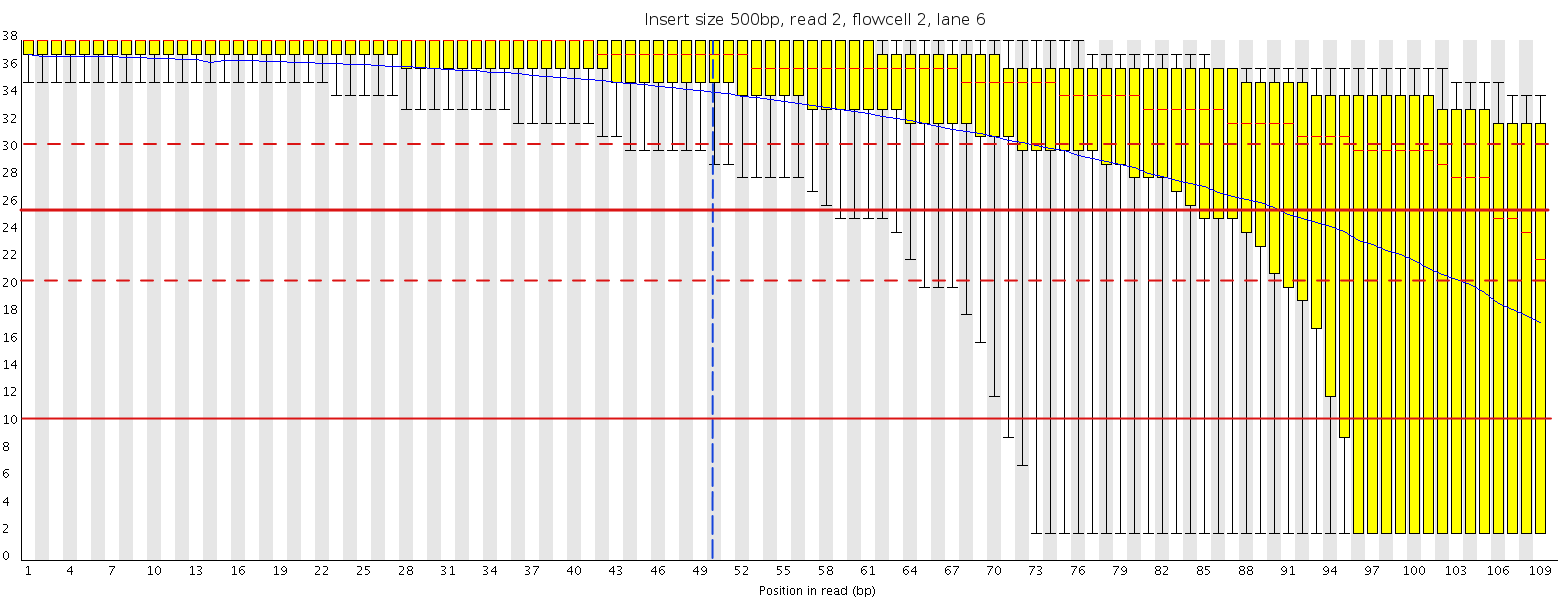

Supplement: Figure S13 — (PNG) [file pone.0026314.s013.png]

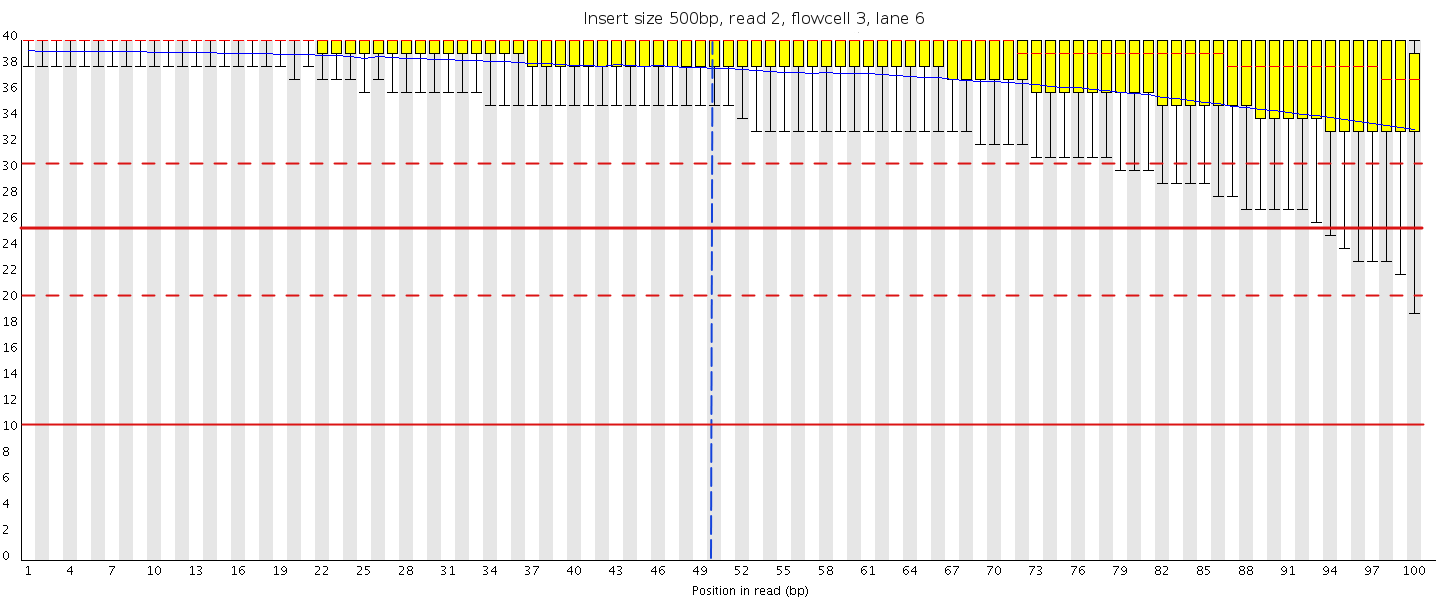

Supplement: Figure S14 — (PNG) [file pone.0026314.s014.png]

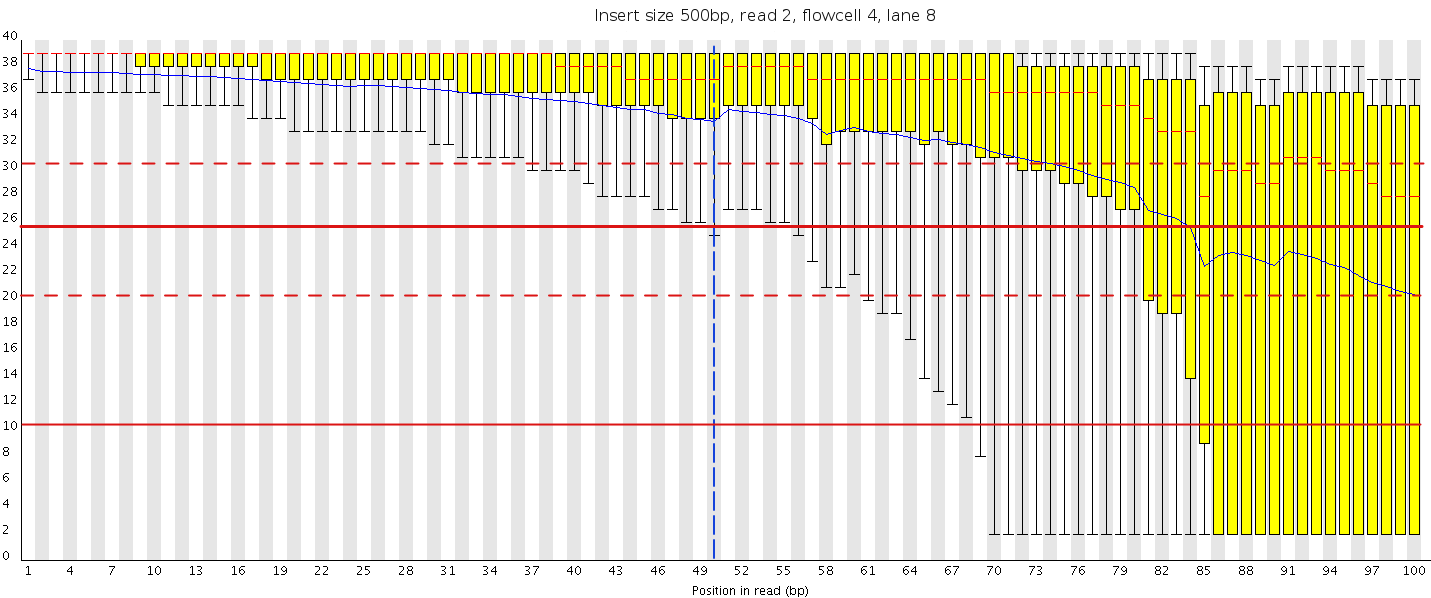

Supplement: Figure S15 — (PNG) [file pone.0026314.s015.png]

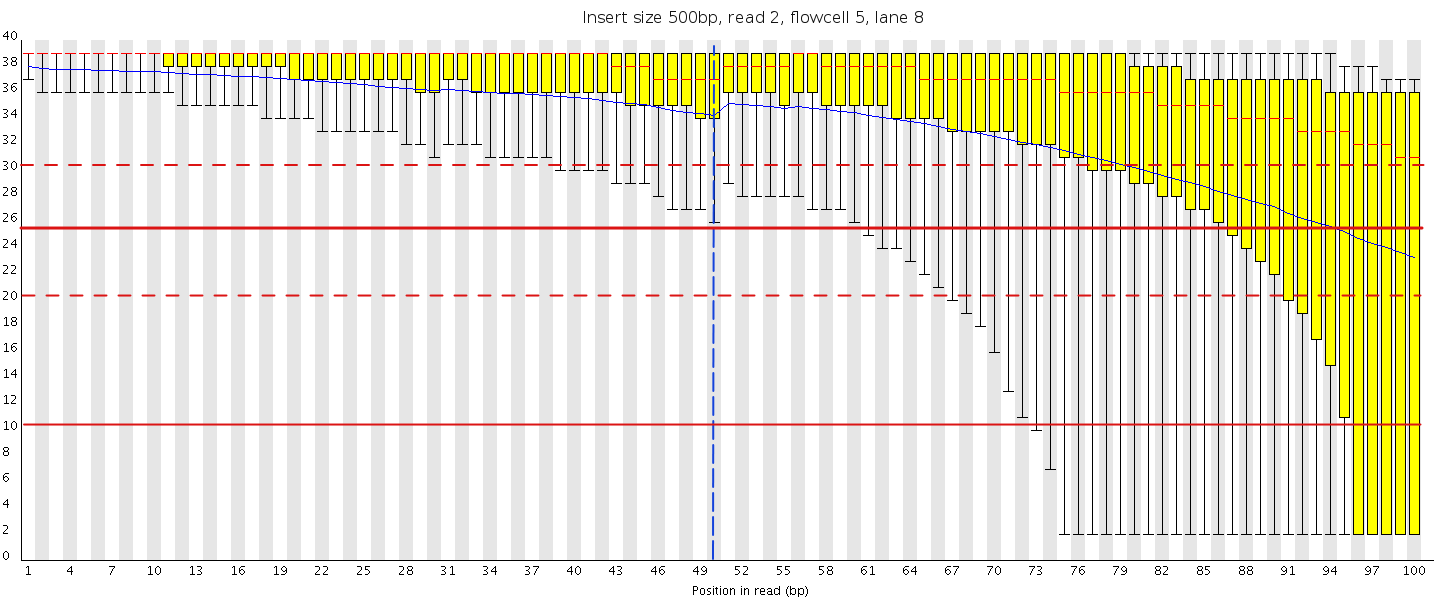

Supplement: Figure S16 — (PNG) [file pone.0026314.s016.png]

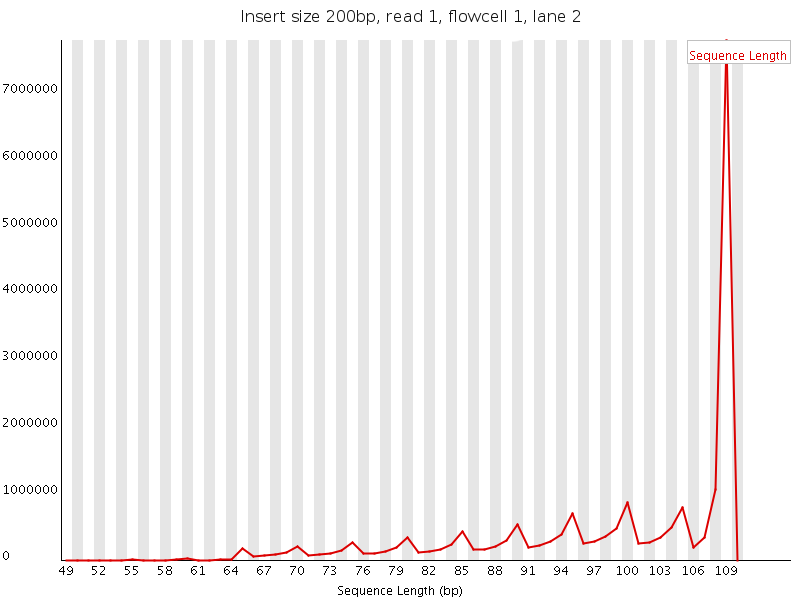

Supplement: Figure S17 — Example length distribution after trimming. Example of read length distribution for the filtered data set corresponding to Figure S3. A majority of the reads are kept at full length. The wave-like pattern in cycles of 5 bp comes from that nH is set to 5. (PNG) [file pone.0026314.s017.png]
