# Supplementary material for: ConDeTri - A Content Dependent Read Trimmer for Illumina Data
Source: PLoS One. 2011 Oct 19;6(10):e26314. doi: 10.1371/journal.pone.0026314 (PMC3198461; doi:10.1371/journal.pone.0026314)
Supplement: Figure S2 — Examples of read trimming. Two examples of read trimming using ConDeTri. (PDF) [file pone.0026314.s002.pdf]

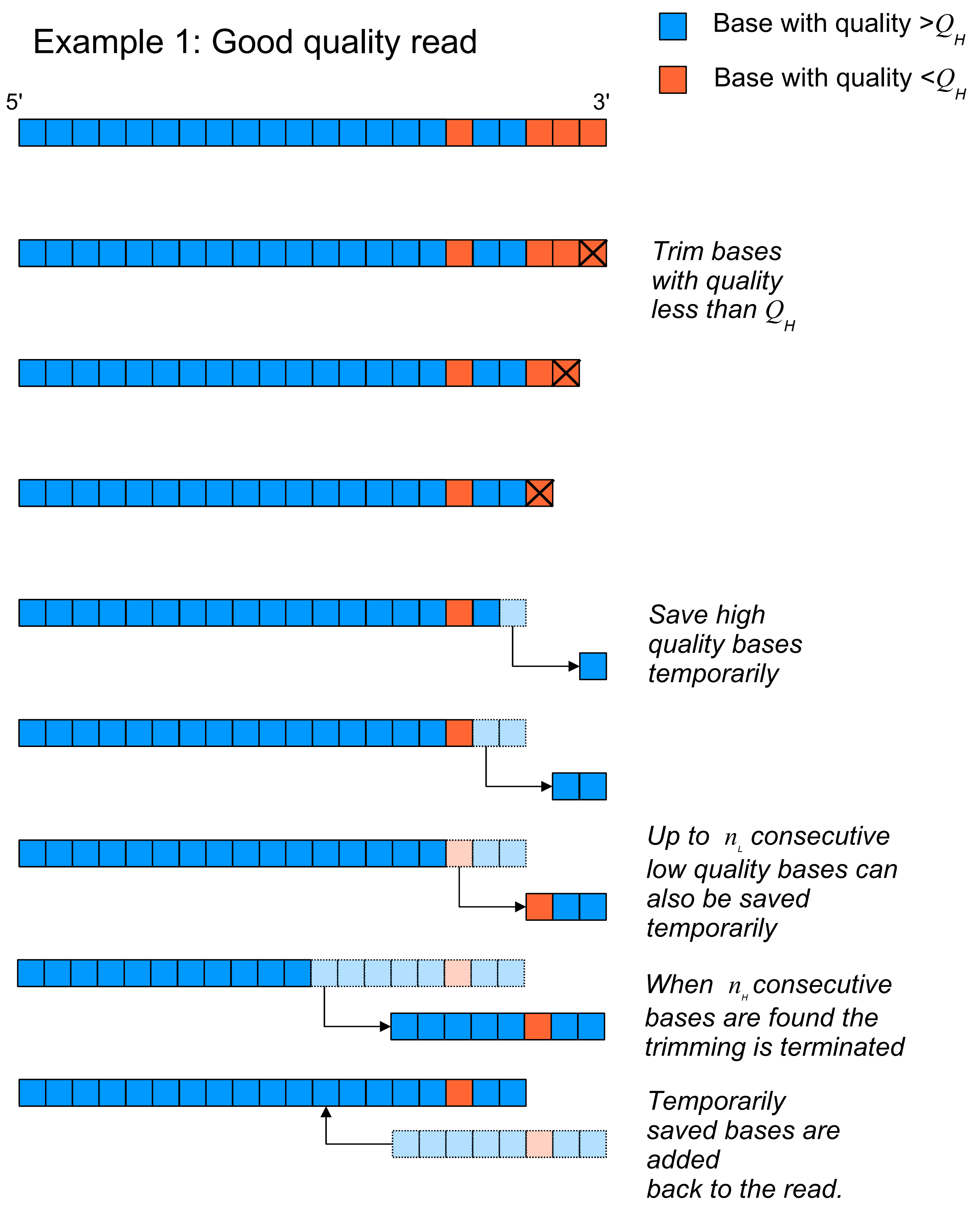

Example 1: Good quality read
Base with quality >Q_H
Base with quality <Q_H
5'
3'
Trim bases with quality less than Q_H
Save high quality bases temporarily
Up to n_L consecutive low quality bases can also be saved temporarily
When n_H consecutive bases are found the trimming is terminated
Temporarily saved bases are added back to the read.

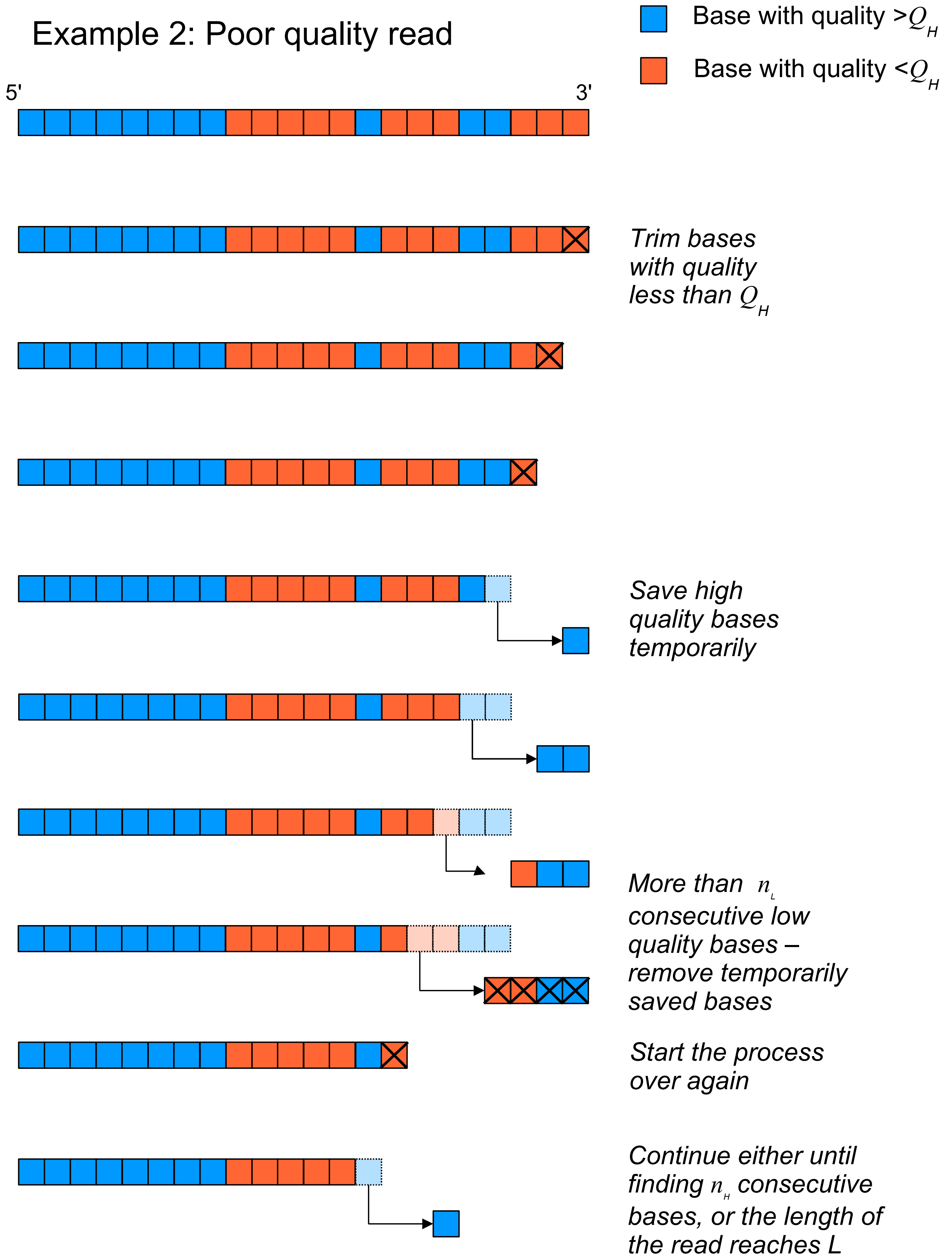
Example 2: Poor quality read
Base with quality >Q_H
Base with quality <Q_H
5'
3'
Trim bases with quality less than Q_H
Save high quality bases temporarily
More than n_L consecutive low quality bases – remove temporarily saved bases
Start the process over again
Continue either until finding n_H consecutive bases, or the length of the read reaches L
