# Supplementary material for: ConDeTri - A Content Dependent Read Trimmer for Illumina Data
Source: PLoS One. 2011 Oct 19;6(10):e26314. doi: 10.1371/journal.pone.0026314 (PMC3198461; doi:10.1371/journal.pone.0026314)
Supplement: Table S1 — Data before and after filtering. The number of reads before and after filtering for the data used for estimating ConDeTri default parameters. (PDF) [file pone.0026314.s018.pdf]

**Data set**	**Reads before filtering**	**Read after filtering**	**% kept**
Insert size 200, flowcell 1, lane 2	54,432,098	41,751,678	77%
Insert size 300, flowcell 1, lane 6	40,332,714	33,238,938	82%
Insert size 400, flowcell 2, lane 2	48,932,200	28,468,594	58%
Insert size 500, flowcell 2, lane 6	41,955,926	26,519,064	63%
Insert size 500, flowcell 3, lane 6	33,640,566	29,484,686	88%
Insert size 500, flowcell 4, lane 8	55,992,656	39,857,038	71%
Insert size 500, flowcell 5, lane 8	84,748,438	64,803,662	76%
